# Deepwater Horizon oil spill impacts on sea turtles could span the Atlantic

**DOI:** 10.1098/rsbl.2015.0596

**Published:** 2015-12

**Authors:** Nathan F. Putman, F. Alberto Abreu-Grobois, Iñaky Iturbe-Darkistade, Emily M. Putman, Paul M. Richards, Philippe Verley

**Affiliations:** 1 Cooperative Institute for Marine and Atmospheric Studies, Rosenstiel School for Marine and Atmospheric Science, University of Miami, 4600 Rickenbacker Causeway, Miami, FL 33149, USA; 2 Unidad Académica Mazatlán, Instituto de Ciencias del Mar y Limnología, Universidad Nacional Autónoma de México, Mazatlán, Sinaloa 82040, Mexico; 3 1252 South Alhambra Circle, Apt. 4, Coral Gables, FL 33146, USA; 4 National Marine Fisheries Service, Southeast Fisheries Science Center, 75 Virginia Beach Drive, Miami, FL 33149, USA; 5 Institut de Recherche pour le Développement (IRD), UMR 248 MARBEC, Centre de Recherche Halieutique Méditerranéenne et Tropicale, av. Jean Monnet, B.P. 171, Sète cedex 34203, France

**Keywords:** ocean-circulation model, sea turtle, oil spill, movement ecology, distribution

## Abstract

We investigated the extent that the 2010 Deepwater Horizon oil spill potentially affected oceanic-stage sea turtles from populations across the Atlantic. Within an ocean-circulation model, particles were backtracked from the Gulf of Mexico spill site to determine the probability of young turtles arriving in this area from major nesting beaches. The abundance of turtles in the vicinity of the oil spill was derived by forward-tracking particles from focal beaches and integrating population size, oceanic-stage duration and stage-specific survival rates. Simulations indicated that 321 401 (66 199–397 864) green (*Chelonia mydas*), loggerhead (*Caretta caretta*) and Kemp's ridley (*Lepidochelys kempii*) turtles were likely within the spill site. These predictions compared favourably with estimates from in-water observations recently made available to the public (though our initial predictions for Kemp's ridley were substantially lower than in-water estimates, better agreement was obtained with modifications to mimic behaviour of young Kemp's ridley turtles in the northern Gulf). Simulations predicted 75.2% (71.9–76.3%) of turtles came from Mexico, 14.8% (11–18%) from Costa Rica, 5.9% (4.8–7.9%) from countries in northern South America, 3.4% (2.4–3.5%) from the United States and 1.6% (0.6–2.0%) from West African countries. Thus, the spill's impacts may extend far beyond the current focus on the northern Gulf of Mexico.

## 1. Background

In earlier papers, we advocated a ‘movement ecology approach’ to predict spatio-temporal variation in distribution of cryptic and difficult to sample life-stages [1,2]. We proposed that the distribution of juvenile sea turtles could be estimated by initiating simulations of hatchling sea turtle movement from nesting beaches (locations of known occurrence and abundance) within a high-resolution ocean-circulation model [1,2]. Variations on this approach have been used to examine a number of questions in sea turtle biology, in which a general depiction of the distribution of the oceanic life-stage is required (e.g. [3,4]). In principle, this approach can also provide specific estimates of turtle distribution at precise areas and times—as would be desirable, for instance, to investigate the number of turtles in the vicinity of seasonally operating fisheries or at sites of marine energy development.

The 2010 Deepwater Horizon oil spill was the largest in US history and its harm to wildlife, including sea turtles, captured the attention of the world [5]. Recently, the official damage assessment for oceanic-stage turtles was released to the public [6]. We used this opportunity to compare whether predictions of the ‘null hypothesis’ of turtle distribution, as estimated from simulations of passive drift via ocean currents and demographic information (e.g. starting population size and stage-specific survival rates), provide results similar to those obtained from in-water observations [6]. We then attempted to reconcile any major disagreement by simple modifications to the model to account for turtle behaviour.

## 2. Analyses

**Figure 1.**
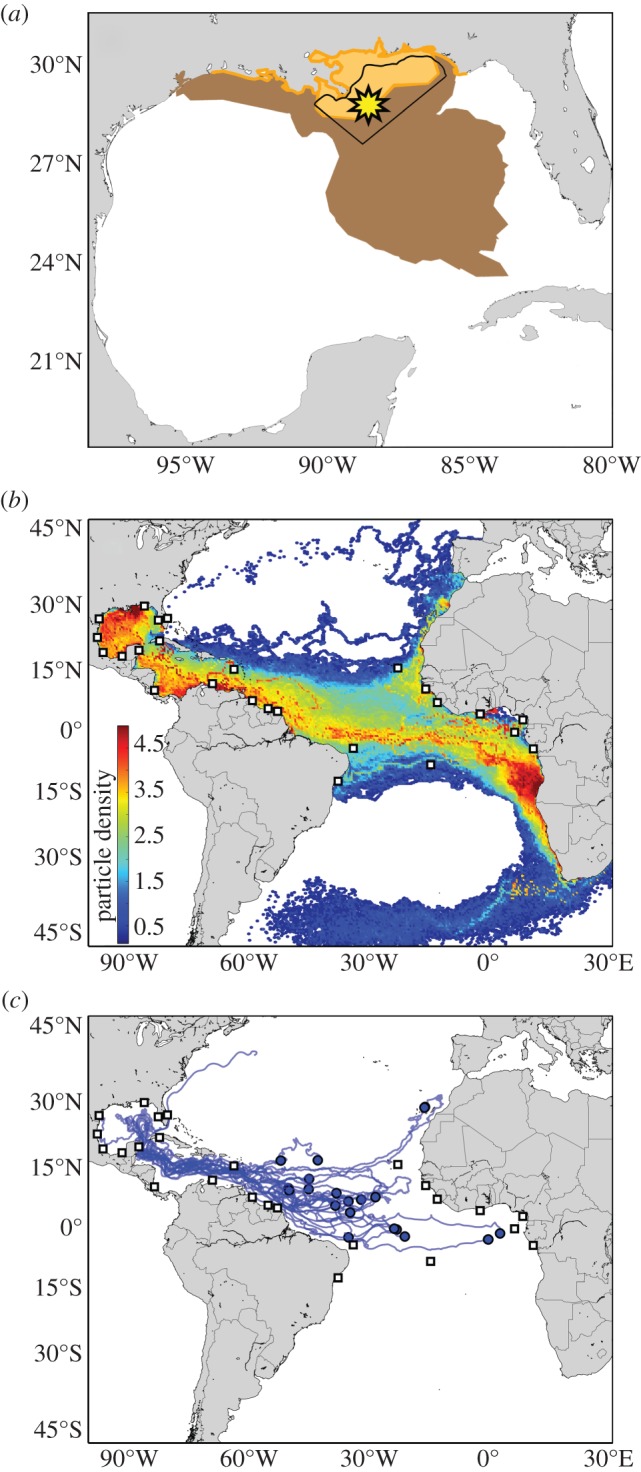
Oceanic connectivity from major turtle nesting beaches to the Deepwater Horizon oil spill site. (*a*) The yellow star indicates the location of the Deepwater Horizon rig, brown shading the potential extent of oil from the Deepwater Horizon Trajectory Map Archive, and orange shading the areas where turtles were observed (http://gomex.erma.noaa.gov/). The black line denotes the area of the oil spill used in analyses. (*b*) Predicted distribution of 154 000 particles backtracked from the spill site throughout the 5-year simulation. Colours indicate particle density by grid cell (counted daily, log_10_-scaled) and thus relative likelihood of transport into the spill site from a given location. White squares show sea turtle nesting beaches considered in our analyses. (*c*) Trajectories of drifters deployed east of 50°W (blue circles) that reached the Gulf of Mexico in less than 2 years during the years 2003–2013 (http://www.aoml.noaa.gov/phod/dac/index.php).

Full details on the methods and data used in analyses are provided in the electronic supplementary material. Transport from sea turtle nesting beaches to the site of the Deepwater Horizon oil spill was estimated from particle-tracking simulations within hindcast output from the Global Hybrid Coordinate Ocean Model (HYCOM) [2]. The spill area used in our analyses encompassed the cumulative surface oil map layer from the National Oceanic and Atmospheric Administration (NOAA) Environmental Response Management Application, but was conservative with respect to the entire potential oil footprint (figure 1*a*). Within this area, 1000 virtual particles were released daily at random locations between April and August 2010, coinciding with the time of the spill. Particles were backtracked for 5 years to determine where a particle came from to reach its final position at the spill site (figure 1*b*,*c*). We recorded the number of particles that passed within approximately 50 km of major green (*Chelonia mydas*), loggerhead (*Caretta caretta*) and Kemp's ridley (*Lepidochelys kempii*) nesting beaches throughout the Atlantic Basin (the three turtle species most abundant in in-water surveys). Transport predictions were weighted by population size (electronic supplementary material, table S1) to assess the proportion of turtles entering the spill site from each population and cohort of oceanic-stage turtles (as in previous models of green and loggerhead turtle dispersal, we considered ages 0–5 years [2,7], and for Kemp's ridley, 0–2 years [1]).

The population of each species/cohort contributing the most particles to the spill was selected as a ‘focal’ population. From that location, 1000 particles were released daily during the population's 75 days of peak hatchling emergence. Particles were forward-tracked and the percentage entering the spill site from April through August 2010 was recorded. The number of turtles of each cohort from the focal population at the spill site was calculated by multiplying estimates of hatchling abundance, annual survival for each year at sea and the percentage of forward-tracked particles arriving at the spill site. Abundance estimates for each remaining population were inferred by multiplying its proportional contribution derived from backtracking simulations by the number estimated for the focal population. To bracket uncertainty in oceanic-stage survival, we performed calculations using the median (81.7%), minimum (25%) and maximum (94%) published values for annual survival in the Atlantic Basin (electronic supplementary material, table S2). In these initial simulations, we assumed passive drift of particles. Directional swimming, even by small turtles, can impact their oceanic movements [7–9] but is not described well enough for all populations considered to accurately parametrize in our model. This issue remains important to resolve and is revisited below.

### (a) Predictions of abundance

**Figure 2.**
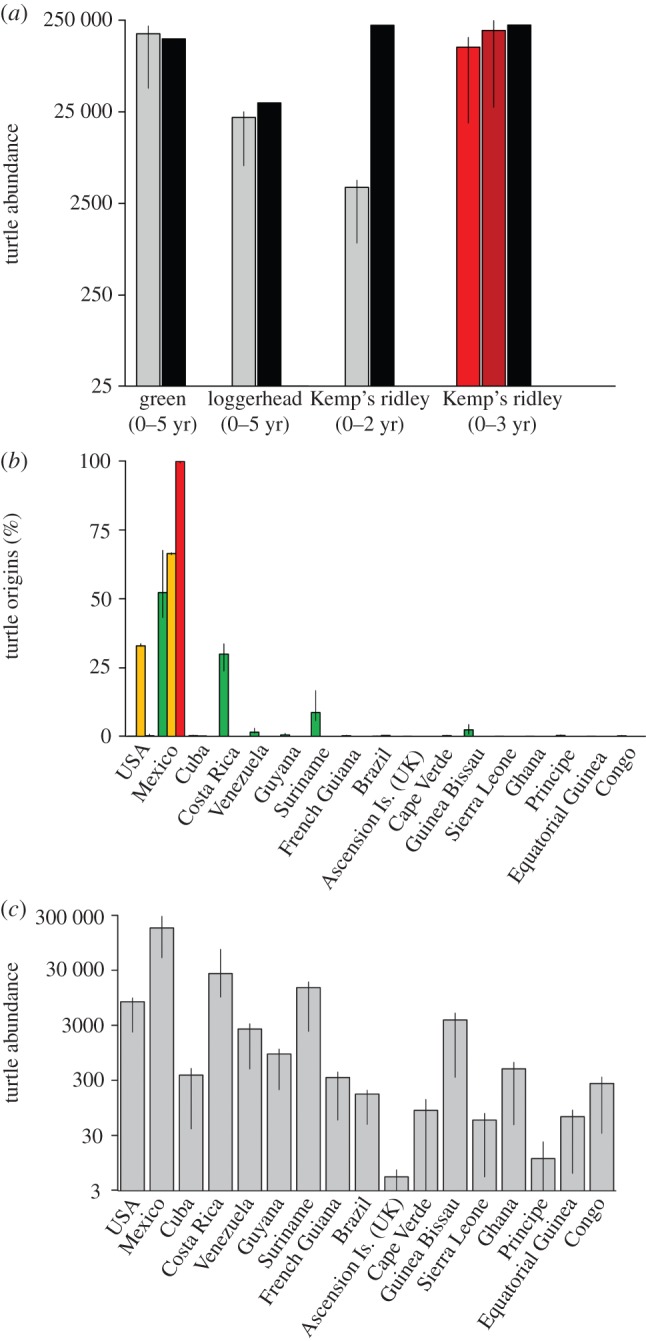
Predictions of turtle abundance and population sources at the spill site. (*a*) Grey bars show turtle abundance predicted by passive transport simulations. Red bars are results for Kemp's ridley simulations depicting ‘retentive behaviour’ within the spill site (lighter red) and across the northeastern Gulf of Mexico (darker red). Error bars indicate results obtained when using the minimum and maximum values of oceanic-stage survival. The black bar is the estimate from in-water observations [6]. (*b*) Percentage of green (green), loggerhead (yellow) and Kemp's ridley (red) turtles at the spill site by country of origin (error bars as in *a*). (*c*) Predicted total turtle abundance at the spill site by country of origin (error bars as in *a*).

Simulations predicted 175 064 (range = 41 313–213 248) green, 21 363 (range = 6349–24 646) loggerhead and 3693 (range = 908–4430) Kemp's ridley turtles present in the vicinity of the spill site. From in-water observations, Wallace *et al*. estimated 154 000 green, 30 800 loggerhead and 217 000 Kemp's ridley oceanic-stage turtles (N.B., no estimate of uncertainty was reported) [6]. While our model agrees well with the in-water estimates for green and loggerhead turtles, the Kemp's ridley estimates differ by two orders of magnitude (figure 2). We therefore examined whether incorporating simple modifications to account for the behaviour of juvenile Kemp's ridley within the simulations would yield better agreement between approaches.

A recent study designed to extract swimming behaviour from the tracks of oceanic-stage Kemp's ridley and green turtles in the eastern Gulf of Mexico showed that directed swimming played an important role in the movement of both species [9]. The swimming orientation of green turtles suggested many transited relatively quickly through this region, whereas orientation of Kemp's ridley turtles appeared to promote their retention within the northeastern Gulf of Mexico [9]. We therefore incorporated the ‘retentive behaviour’ of Kemp's ridley turtles in another set of forward-tracking simulations.

We forward-tracked 9000 particles from the three major Kemp's ridley nesting regions (Tamaulipas, Mexico; Veracruz, Mexico; Texas, USA [1]) during the three months of hatchling emergence (June, July and August), assuming a 48 h ‘frenzy period’ during which turtles swam offshore at 0.25 m s^−1^ followed by 2 years of passive drift. Simulations were performed for the 2007, 2008, 2009 and 2010 cohorts within the Gulf of Mexico HYCOM [1]. Turtles from the 2007 cohort (not yet 3 years old at the time of the spill) were included in these simulations to account for uncertainty as to when the transition from oceanic to near shore habitats occurs. For this cohort, particles stopped moving after the second year and third-year survival was set to 50%. To depict likely ‘retentive behaviour’ [9], any particle that entered the previously defined spill area prior to 31 August 2010 was assumed to remain there (though still subject to survival rates described previously). Separately, we counted particles that crossed north of 28°N between the western edge of Louisiana and the Florida Panhandle prior to 31 August 2010 to test, at a regional scale, whether the model of ‘retentive behaviour’ was consistent with in-water estimates of Kemp's ridley abundance.

Simulations mimicking ‘retentive behaviour’ predicted 124 973 (range = 18 537–159 861) Kemp's ridley turtles within the spill site and 190 194 (range = 27 584–243 804) across the northeastern Gulf of Mexico. These predictions (particularly towards the higher end of the range) correspond better to the 217 990 zero- to three-year-old Kemp's ridley turtles estimated from in-water observations [6].

### (b) Predictions of source populations

Green turtles were predicted to originate primarily from Mexico (range = 43.1–67.5%), Costa Rica (range = 23.7–33.6%), Suriname (range = 5.7–16.6%) and Guinea Bissau (range = 0.8–4.3%). Loggerhead turtles were mostly from Mexico (range = 66.0–66.6%) and the United States (range = 32.7–33.6%). Nearly all Kemp's ridley turtles were predicted to be from Mexico (more than 99%). The remaining contributions spanned countries across a wide swathe of the Atlantic (figures 1*b* and 2*b*).

Though the in-water data provide no information with which to compare our predictions of source populations [6], our predictions of turtle movement from distant beaches to the spill site are consistent with other ocean models [4] and observations of surface transport. Surface drifters move from the eastern to western Atlantic [10,11] (figure 1*c*) and *Sargassum* algae (habitat of juvenile turtles [12]) drifts from South American waters into the Caribbean and Gulf of Mexico [13]. Moreover, genetic surveys of green turtles (the species in our simulations with the greatest contributions from distant nesting sites) show that haplotypes endemic to nesting beaches east of 34°W (i.e. the eastern tip of South America) are found at foraging grounds in the northwest Atlantic [2,11], approximately 20% of the Guinea Bissau green turtle population is estimated to use foraging grounds in the northwest Atlantic (spanning Barbados to North Carolina) [11], and juvenile green turtles travel from the Caribbean to the Texas coast [14].

## 3. Implications

Owing to the simplifying assumptions within our model, related to turtle behaviour and the definition of the spill area, our results are presented to spur additional research on turtle populations potentially impacted by the oil spill rather than to make a damage assessment. However, it is noteworthy that the simulations yield abundance estimates comparable with those derived from in-water surveys. Therefore, this modelling technique appears to be sufficiently robust to address questions of sea turtle distribution and abundance across a wide range of spatio-temporal scales—even in situations such as this, where *in situ* data to constrain the model are limited [5].

The major differences between predictions assuming passive drift for Kemp's ridley turtles and predictions in which potential behaviour was simulated, however, imply caution is warranted. Where turtles attempt to remain in, or target, an area, adding a behavioural component to simulations is needed. Even so, much better agreement between the simulations and survey data could be attained, essentially, by coarsening the spatial and temporal resolution of the area of interest. That such modifications were not required for green and loggerhead turtles might suggest that, in regions where turtles are transient, depicting the movement of ocean currents alone can be adequate. Regardless, future work should focus on more realistic representations of turtle behaviour within simulations of movement [7–9].

Our findings provide much-needed geopolitical context for the spill's impacts and a starting point for assessments of the population-level consequences of injuries sustained by oceanic-stage turtles. That more than 95% of turtles were predicted to be from non-US nesting populations suggests deterioration of this previously favourable habitat for young turtles could have lasting implications for turtle populations throughout the Atlantic [15] and highlights the need to include international stakeholders (e.g. governments that have invested in sea turtle conservation) in discussions of restoration (figures 1*b* and 2*c*). Finally, our results call into question the myopic focus on the northern Gulf of Mexico [16] and serve as a reminder that seemingly ‘local’ disturbances in the marine environment can result in impacts that span an extraordinary scale.

## Supplementary Material

Supplemental Information and Supplemental Tables

## Data accessibility

Datasets supporting this article have been uploaded as part of the electronic supplementary material and are deposited in the Dryad Digital Repository (http://dx.doi.org/10.5061/dryad.16q70).
