# Supplementary material for: Deepwater Horizon oil spill impacts on sea turtles could span the Atlantic
Source: Biol Lett. 2015 Dec;11(12):20150596. doi: 10.1098/rsbl.2015.0596 (PMC4707693; doi:10.1098/rsbl.2015.0596)
Supplement: Supplemental Information and Supplemental Tables [file rsbl20150596supp1.pdf]

SUPPLEMENTAL INFORMATION:

Deepwater Horizon Oil Spill Impacts to Sea Turtles Could Span the Atlantic

Nathan F. Putman, F. Alberto Abreu-Grobois, Iñaky Iturbe-Darkistade,

Emily M. Putman, Paul M. Richards, Philippe Verley

Supplemental Methods

Information on the distribution of marine organisms is essential for the conservation and management of resources but is limited by the vast and inaccessible oceanic environment [S1-S3]. In the present case, acquiring information is further hampered due to minimal data for the area prior to the spill and the necessarily strict confidentiality of data collected during the spill as it relates to ongoing litigation [S4]. We circumnavigate the paucity of *in situ* data by applying a novel and widely-applicable method that derives the number and sources of pelagic organisms across any area and time of interest by coupling transport predictions within an ocean circulation model and demographic data. Here, we describe this approach to quantify the number of surface-pelagic stage (also known as oceanic-stage) sea turtles and their source populations in the immediate vicinity of the spill site.

Estimating Ocean Transport

The Deepwater Horizon oil spill began on April 20, 2010 and reports from the Deepwater Horizon Trajectory Archive indicated that the presence of recoverable oil at the ocean surface was observed through July 31, though the possibility of scattered tar balls extended through the final report on August 23 (http://archive.orr.noaa.gov/). We chose the period from March 30 to August 31, 2010 for our analyses to account for turtles that were in the area a few weeks prior to the spill and a few weeks after the perceived threat of oil had dissipated. This choice is likely conservative with respect to sea turtle strandings associated with the oil spill, which were recorded from April 26 to October 20 (though it is noted that a “visibly oiled” turtle found was

dead in Florida on October 30, http://www.nmfs.noaa.gov/pr/health/oilspill/turtles.htm). Had we extended our simulations later into 2010 it is conceivable that we would have detected the presence of young-of-the-year turtles from the 2010 cohort. (In our present analysis, however, none of this age class were predicted to be at the spill site.)

The early years of most sea turtle species are characterized by an oceanic stage of dispersal via ocean currents [S1]. Transport from sea turtle nesting beaches to the site of the Deepwater Horizon oil spill was thus estimated using hindcast output from the Global Hybrid Coordinate Ocean Model (HYCOM). HYCOM output is a daily snapshot of surface velocity at a spatial resolution of 0.08° (approximately 6-9 km grid spacing), HYCOM assimilates satellite and *in situ* measurements of ocean velocity and temperature and is widely used to generate realistic estimates of ocean transport [S5,S6]. Dispersal simulations were performed with ICHTHYOP v. 2 particle tracking software [S7]. We released 1000 virtual particles each day from March 30 to August 31, 2010 (a total of 154,000 particles). Particles were randomly released across a polygon within the oil spill site that was defined using publicly available charts from the NOAA Environmental Response Management Application (ERMA) (http://gomex.erma.noaa.gov/erma.html). For simplicity, and to be conservative with respect to our estimate, the polygon used was smaller than the total extent of surface oil with vertices at 29.2°N, 90.8°W; 27.51°N, 88.53°W; 30.08°N, 85.0°W; 30.9°N, 88.0°W. Particles were released in the surface layer of HYCOM in water depths greater than 30 m (Fig. 1a). For advection of particles, ICHTHYOP implemented a Runge-Kutta 4th order time-stepping method whereby a particle's "previous" position was calculated at half-hour intervals for a total of 5 years. To simulate sub-grid scale turbulent processes, horizontal dispersion was also included in the advection process [S7]. Thus, ICHTHYOP's backtracking routine determined where a particle came from to reach its final location at the spill site.

We qualitatively compared transport predictions to Lagrangian drifter data from the Global Drifter Program (http://www.aoml.noaa.gov/). In particular, we were interested in whether there was empirical evidence for oceanic connectivity between distant sea turtle nesting beaches and the Deepwater Horizon oil spill site. We identified drifters in the AOML drifter database that reached the Gulf of Mexico (north of 18°N, west of 80°W) from deployment sites east of 50°W within less than 2 years during the years 2003-2013.

Estimating Turtles Encountering the Oil Spill Site

In backtracking simulations, ICHTHYOP recorded the number of particles released from the spill site that passed within the vicinity (~50 km) of major sea turtle nesting beaches throughout the Atlantic Basin (24 green, 8 loggerhead, and 4 Kemp's ridley nesting beaches). The number of particles entering each nesting area was divided by the total number of particles entering all nesting areas. Following the methods of Putman and Naro-Maciel [S1], the results of the particle-tracking experiments were weighted by nesting beach population size (in this case, number of hatchlings produced). These computations were performed at yearly intervals to assess the proportion of juvenile turtles (0 – 2 years for Kemp's ridley, 0 – 5 years for green and loggerhead) entering the spill site from each population and cohort (age class). Nesting beaches included: SE Florida (USA); SW Florida (USA); NW Florida (USA); Texas (USA); Tamaulipas (Mexico); Veracruz (Mexico) ; Campeche (Mexico); Quintana Roo (Mexico) ; Cuba ; Costa Rica; Venezuela; Aves Island (Venezuela); Guyana; Suriname; French Guiana; Atol das Rocas (Brazil); Bahia/Sergipe (Brazil); Ascension Island (UK); Cape Verde Islands; Guinea Bissau; Sierra Leone; Ghana; São Tomé and Príncipe; Bioko Island (Equatorial Guinea); and the Congo.

To estimate the number of juvenile turtles that entered the oil spill site, we further examined transport predictions from the nesting beaches that were identified as contributing the largest number of turtles to the spill site for each species and cohort. In these simulations, we released 1000 virtual particles per day in the vicinity of the nesting beach during the 75 days of peak hatchling emergence. Particles were tracked forwards through time and the percentage entering the spill site between March 30, 2010 and August 31, 2010 was recorded. This allowed for the calculation of the number of turtles from the focal population at the spill site:

$$\{1\}\ Tf = H * F * So^{Y}$$

Where H is the number of hatchlings produced by the focal population, F is the percentage of forward-tracked particles arriving to the spill site, So is annual percent oceanic survival, and Y is the age class of animals (i.e., the number of years at sea). Multiplied together they produce, Tf, the number of individuals of a given cohort from the focal population at the spill site.

Upon obtaining the number of individuals for a single population we quantified the number of individuals arriving from each of the other (non-focal) populations by applying the following relationship:

$$\{2\}\ Tn = (Tf / Bf\ ) * Bn$$

Where *Tf* is the number of individuals at the spill site of a given cohort from the focal population, *Bf* is the relative proportion of particles arriving to the spill site from the focal population that was estimated from backtracking simulations, and *Bn* is the relative proportion of particles arriving to the spill from the non-focal population that was estimated from backtracking simulations. This yields *Tn*, the number of individuals of a given cohort from the non-focal population at the spill site.

From these equations we calculated the number of turtles at the spill site from each species, population, and cohort (age-class). This allowed us to quantify the number of turtles predicted to be from each country and its relative contribution to turtles at the spill site. Further, we computed the percentage of the total number of turtles from a given population and cohort that were still alive at the end of the simulation. For example, a population predicted to have 2 turtles at the spill site out of 1000 surviving turtles would have 0.2% of its population at the spill site, whereas a population predicted to have 2 turtles at the spill site out of 10 surviving turtles would have 20% of its population at the spill site.

Demographic parameters

Estimates of population size were based on hatchling production, as this is the most relevant metric for our analyses of oceanic-stage turtle transport to the oil spill. We had a hierarchy of four tiers for setting the initial population size: (1) direct counts of hatchlings released from the nesting beach, (2) annual counts of nests from the nesting beach, (3) female population size reported in the latest status review of the green sea turtle [S8], and (4) nest counts reported in the State of the World's Sea Turtles (SWOT) Reports, volumes 2, 5, and 6 [S9-S11].

The best data were available for Kemp's ridley beaches where hatchlings released in Tamaulipas, Mexico [S12] and Texas, USA [S13] and nests deposited in Veracruz, Mexico [S14] have been made available on an annual basis. Thus, annual variability in hatchling production

could be factored in to analyses for this species. This was rarely the case for green and loggerhead turtles, thus where a time series of data was available for these species we simply averaged the values between 2010 and 2006, the most relevant time period. For green turtle beaches with data from the 2015 status review [S8], we used the following equation to calculate the number of nests:

$$\{3\}\ N = (P_f\ / R) * C_{freq.}$$

Where P_f is the female population size, R is the remigration interval (years between nesting), $C_{freq.}$ is clutch frequency (the number of nests deposited per year by an individual) and N is the number of nests deposited. If a given population did not have an estimated remigration interval or clutch frequency the mean values of 2.5 years and 3 nests per year were used [S12]. For both the number of nests calculated [S8] and nest counts directly reported [S9-S11,S14], we converted the number of nests (N) to hatchlings released (H) by,

$$\{4\}\ H = N * C_{size} * C_{surv.}$$

Where C_{size} is the clutch size (number of eggs per nest) for each species, and C_{surv} is the clutch survival for each species. Clutch size and survival were determined by a search through the relevant literature [S15-S23] and selecting the median value for each parameter (see also Excel Tables S1, S9). For Kemp's ridley turtles, we assumed 110 eggs per clutch and clutch survival of 63%. For green turtles we assumed 118.7 eggs per nest and clutch survival of 72.5%. For loggerhead turtles we assumed 116.8 eggs per nest and clutch survival of 72%.

Though central to calculating the number of turtles arriving to the spill site (see equation {1}), considerable uncertainty surrounds estimates of oceanic-stage survival. Due to limited data, we pooled survival estimates within the Atlantic basin (including Gulf of Mexico and Mediterranean Sea) for green, loggerhead, and Kemp's ridley turtles. These values include field-based estimates and derivations from demographic models / life-tables [S20-S28]. To bracket the uncertainty in this parameter performed calculations with equation {1} using the median (81.7%), minimum (25%), and maximum (94%) values obtained from the literature.

Comparison to estimates of abundance from in-water surveys

A draft of a technical report titled “Estimating degree of oiling of sea turtles and surface habitat during the Deepwater Horizon oil spill: implications for injury quantification” was published on September 3, 2015 (https://pub-dwhdatadiver.orr.noaa.gov/dwh-ar-documents/894/DWH-AR0279127.pdf) [S29]. Wallace et al. [S29] extrapolate data collected during in-water sea turtle rescue operations to estimate the total number of turtles in the vicinity of the spill site by species (Kemp’s ridley loggerhead, green, hawksbill, and unidentified). They further estimate to what extent the turtles present would have been directly exposed to oil and died. Our interest was in the total number of Kemp’s ridley, loggerhead, and green turtles in the area, as this is what our model was designed to predict. We took data from Table 3 (p. 35) to compare predictions from the model described above. Additionally, we took data from and Table 4 (p. 36) for age 3 year Kemp’s ridley turtles to add to total of oceanic juveniles for comparison to the simulations described below (in which ages 0-3 years were modeled).

Simulating “Retentive Behavior” in Kemp’s Ridley Turtles

In comparison to in-water estimates of abundance [S29], predictions from the model described above agreed reasonably well for green and loggerhead turtles. However, the Kemp’s ridley estimates differed by 2 orders of magnitude. This major discrepancy suggest that either, (1) the model, as parameterized above, is inadequate to estimate the in-water abundance of Kemp’s ridley in the northern Gulf of Mexico or (2) the in-water estimates for Kemp’s ridley are wrong. The second possibility is outside of the scope of this paper, though certainly a number of un-tested assumptions go into extrapolating in-water count data to regional abundance [S29]. In any case, the two major sources of uncertainty in our model are oceanic-stage survival and swimming behavior in turtles. However, given that even calculating abundance with the highest value of annual survival (94%) did not appreciably improve agreement between model predictions and [S29], we decided to examine whether incorporating simple modifications to account for the behavior of juvenile Kemp’s ridley within the simulations would yield better agreement between approaches.

Though data on the swimming behavior of oceanic-stage turtles is very limited, a recently published study tracking oceanic-stage Kemp’s ridley (and green) turtles in the eastern Gulf of

Mexico provides valuable clues. The study was designed to extract swimming behavior from the tracks of oceanic-stage turtles and clearly showed that directed swimming played an important role in the net movement of both species [S30]. The swimming orientation of green turtles suggested many transited relatively quickly through this region, whereas orientation of Kemp's ridley turtles appeared to promote their retention within the northeastern Gulf of Mexico. We therefore incorporated the "retentive behavior" of Kemp's ridley turtles in another set of forward-tracking simulations. Modeling such behavior could be done in several ways [e.g., S31-S33], in this instance, we opted for a simplistic approach.

We forward-tracked 9000 particles from the 3 major Kemp's ridley nesting regions (Tamaulipas, Mexico; Veracruz, Mexico; Texas, USA) during the 3 months of hatchling emergence (June, July, and August), assuming a 48 h "frenzy period" during which turtles swam offshore at 0.25 m/s followed by 2 years of passive drift [S34]. Simulations were performed for the 2007, 2008, 2009, and 2010 cohorts within hindcast output from the Gulf of Mexico HYCOM. Like Global HYCOM, Gulf of Mexico HYCOM has a daily snapshot of surface velocity but is at finer a resolution of 0.04° rather than 0.08° [S34]. The number of turtles from each region was calculated for each cohort following equation {1}, above, using the number of hatchlings released in a given year and the median, minimum, and maximum oceanic-stage survival values. Turtles from the 2007 cohort (not yet 3 years old at the time of the spill) were included in these simulations to account for uncertainty as to when the transition from surface-pelagic to nearshore habitats occurs. For this cohort, particles stopped moving after the second year and third-year survival was set to 50% [S22]. To depict likely "retentive behavior" [9], any particle that entered the previously-defined spill area prior to August 31, 2010 was assumed to remain there (though still subject to survival rates described previously). Separately, we counted particles that crossed north of 28°N between the western edge of Louisiana and the Florida panhandle prior to August 31, 2010 to test, at a regional-scale, whether the model of "retentive behavior" was consistent with in-water estimates of Kemp's ridley abundance. These values were divided by the total number of particles released in a given year (9000) to generate the probability of transport from each beach to these areas (F in equation {1}).

Caveats and Qualifications

There are limitations to our modeling approach that arise due to uncertainty in turtle demographics and behavior. In all cases, however, we attempted to make assumptions conservative with respect to the number of turtles predicted to be in the vicinity of the spill site. For instance, we only considered input to the spill area from major nesting sites with consistently reported nest counts. Excluding minor and poorly known nesting sites results in lower estimates of turtles at the spill. Additionally, dispersal simulations were based only on surface currents due to the uncertainty of orientation behavior in different turtle populations. Inclusion of directed swimming might considerably increase our estimate of turtles at the spill site. When swimming has been simulated in populations where data on such behavior are available (e.g., the offshore swimming "frenzy" of hatchlings [S35] and inherited magnetic navigation instructions that guide turtles' transoceanic migrations [S36]), it greatly increases the proportion of the population that arrive at distant, productive foraging grounds (with environmental characteristics similar to those at the spill site), by orienting into ocean currents that facilitate such transport [S31,S32]. Likewise, these behavior increase survival by reducing the probability of remaining in or encountering nearshore waters where predation risk is highest [S31,S32].

Regardless, our approach brackets a range of possibilities that would otherwise be unknown. Thus, despite some limitations, this method of coupling transport predictions with demographic data provides a broadly-applicable tool to derive the number and sources of pelagic organisms within any area and over any recent period of interest (associated, for instance, with an oil spill, a fishery, or the proposed location of marine energy development). Furthermore, the methods can be easily modified to take advantage of additional information that is available for particular species or populations (e.g., diving behavior, oriented swimming, and duration of pelagic phase). This simple approach will be invaluable to researchers assessing the population-level impacts of anthropogenic disturbances and conducting damage assessments.

Supplemental References

S1. Putman NF, Naro-Maciel E. 2013 Finding the 'lost years' in green turtles: insights from ocean circulation models and genetic analysis. *Proceedings of the Royal Society – B* **280**, 20131468.

S2. Cowen RK, Paris CB, Srinivasan A. 2006 Scaling of connectivity in marine populations. *Science* **311**, 522-527.

S3. Werhner FE, Cowen RK, Paris CB. 2007 Coupled biological and physical models: present capabilities and necessary developments for future studies of population connectivity. *Oceanography* **20**, 54-69.

S4. Bjorndal KA, Bowen BW, Chaloupka M, Crowder LB, Heppell SS, Jones CM, Lutcavage ME, Policansky D, Solow AR, Witherington BE. 2011 Better science needed for restoration in the Gulf of Mexico. *Science* **331**, 537-538.

S5. Chassignet EP, Hurlburt HE, Smedstad OM, Halliwell GR, Hogan PJ, Wallcraft AJ, Baraille R, Bleck R. 2007 The HYCOM (Hybrid Coordinate Ocean Model) data assimilative system. *Journal of Marine Systems* **65**, 60-83.

S6. Putman NF, He R. 2013 Tracking the long-distance dispersal of marine organisms: sensitivity to ocean model resolution. *Journal of the Royal Society Interface* **10**, 20120979.

S7. Lett C, Verley P, Mullon C, Parada C, Brochier T, Penven P, Blanke B. 2008 A Lagrangian tool for modelling ichthyoplankton dynamics. *Environmental Modelling Software* **23**, 1210-1214.

S8. Seminoff, J.A., C.D. Allen, G.H. Balazs, P.H. Dutton, T. Eguchi, H.L. Haas, S.A. Hargrove, M.P. Jensen, D.L. Klemm, A.M. Lauritsen, S.L. MacPherson, P. Opay, E.E. Possardt, S.L. Pultz, E.E. Seney, K.S. Van Houtan, R.S. Waples. 2015. Status Review of the Green Turtle (Chelonia mydas) Under the U.S. Endangered Species Act. NOAA Technical Memorandum, NOAANMFS-SWFSC-539. 571pp.

S9. SWOT Report, Volume II, http://www.seaturtlestatus.org/report/swot-volume-2

S10. SWOT Report, Volume V, http://www.seaturtlestatus.org/report/swot-volume-5

S11. SWOT Report, Volume VI, http://seaturtlestatus.org/report/swot-report-vol-6

S12. Gallaway BJ, Caillouet CW, Plotkin PT, Gazey WJ, Cole JG, Raborn SW. 2013. Kemp's ridley stock assessment project final report. LGL Ecological Research Associates, Inc., Bryan, Texas. Prepared for Gulf States Marine Fisheries Commission, Ocean Springs, Mississippi. 291 pp.

S13. Shaver DJ, Caillouet CW. 2015 Reintroduction of Kemp's ridley (Lepidochelys kempii) sea turtle to Padre Island National Sea Shore, Texas and it's connection to head-starting. *Herpetological Conservation and Biology* **10**, 378-435.

S14. González DMR. (2014). Fortalecimiento del Programa de Protección de Tortuga Marina en el Estado de Veracruz. Programa de Conservación de Especies en Riesgo. PROCER 2014. CONAMP-SEMARNAT. Datos no publicados.

S15. Ackerman RA. 1980 Physiological and ecological aspects of gas exchange by sea turtle eggs. *American Zoologist* **20**, 575-583.

S16. Van Buskrik J, Crowder LB. 1994 Life-history variation in marine turtles. *Copeia* **1994**, 66-81.

S17. Hirth HF. 1980 Some aspects of the nesting behavior and reproductive biology of sea turtles. *American Zoologist* **20**, 507-523.

S18. Fowler LE. 1979 Hatching success and nest predation in the green sea turtle, *Chelonia mydas*, at Tortugero, Costa Rica. *Ecology* **60**, 946-955.

S19. Miller JD, Limpus CJ, Godfrey MH. 2003 Nest site selection, oviposition, eggs, development, hatching and emergence of loggerhead turtles. In *Loggerhead sea turtles* (eds. AB Bolten, BE Witherington) pp. 125-143.

S20. Crouse DT, Crowder LB, Caswell H. 1987 A stage-based population model for loggerhead sea turtles and implications for conservation. *Ecology* **68**, 1412-1423.

S21. Crowder LB, Crouse DT, Heppell SS, Martin TH, 1994. Predicting the impact of turtle excluder devices on loggerhead sea turtle populations. *Ecological Applications* **4**, 437-445.

S22. Heppell SS, Crouse DT, Crowder LB, Epperly SP, Gabriel W, Henwood T, Marquez R, Thompson NB. 2005 A population model to estimate recovery time, population size and management impacts on Kemp's ridley sea turtles. *Chelonian Conservation and Biology* **4**, 767-773.

S23. Turtle Expert Working Group. 2000 Assessment update for the Kemp's ridley and loggerhead sea turtle populations in the western North Atlantic. U.S. Department of Commerce, NOAA Technical Memorandum, NMFS-SEFSC-444 115pp.

S24. Bjorndal KA, Bolten AB, Martins HR. 2003 Estimates of survival probabilities for oceanic-stage loggerhead sea turtles (*Caretta caretta*) in the North Atlantic. *Fishery Bulletin* **101**, 732-736.

S25. Bjorndal KA, Bolten AB, Chaloupka MY. 2003 Survival probability estimates for immature green turtles, *Chelonia mydas*, in the Bahamas. *Marine Ecology Progress Series* **252**, 273-281.

S26. Cassale P, Freggi D, Furii G, Vallini C, Salvemini P, Deflorio M, Totaro G, Raimondi S, Fortuna C, Godley BJ. 2014 Annual survival probabilities of juvenile loggerhead sea turtles indicate high anthropogenic impact on Mediterranean populations. *Aquatic Conservation: Marine and Freshwater Ecosystems,* published online.

S27. Frazer NB. 1986 Survival from egg to adulthood in a declining population of loggerhead turtles, *Caretta caretta*. *Herpetologica* **42**, 47-55.

S28. Sasso CR, Epperly SP. 2007 Survival of pelagic juvenile loggerhead turtles in the open ocean. *Journal of Wildlife Management* **71**, 1830-1835.

S29. Wallace B, Rissing M, Cacela D, Garrison L, McDonald T, Schroeder B, McLamb D, Witherington B, Stacy B. 2015 Estimating degree of oiling of sea turtles and surface habitat during the Deepwater Horizon oil spill: Implications for injury quantification. (ST_TR.02). DWH Sea Turtles NRDA Technical Working Group Report.

S30. Putman NF, Mansfield KM. 2015 Direct evidence of swimming demonstrates active dispersal in the sea turtle "lost years." *Current Biology* 25, 1221-1227.

S31. Putman NF, Veryley P, Shay TJ, Lohmann KJ. 2012 Simulating transoceanic migrations of young loggerhead sea turtles: merging magnetic navigation behavior with an ocean circulation model. *Journal of Experimental Biology* **215**, 863-1870.

S32. Putman NF, Scott R, Verley P, Marsh R, Hays GC. 2012 Natal site and offshore swimming influence fitness and long-distance ocean transport in young sea turtles. *Marine Biology* **159**, 2117-2126.

S33. Scott R, Marsh R, Hays GC. 2012 A little movement orientated to the geomagnetic field makes a big difference in strong flows. *Marine Biology* 159, 481–488

S34. Putman NF, Mansfield KL, He R, Shaver DJ, Verley P. 2013 Predicting the distribution of oceanic-stage Kemp's ridley sea turtles. *Biology Letters* **9**, 20130345.

S35. Wyneken J, Salmon M. 1992 Frenzy and post-frenzy activity in loggerhead, green, and leatherback hatchling sea turtles. *Copeia* **1992**, 478-484.

S36. Lohmann KJ, Putman NF, Lohmann CMF. 2012 The magnetic map of hatchling loggerhead sea turtles. *Current Opinion in Neurobiology* **22**, 336-342.

Supplemental Tables

Description of Supplemental Excel Tables Deposited in the Dryad Digital Repository (http://dx.doi.org/10.5061/dryad.16q70).

Note: tab names are italicized prior to the description of the table.

Table S1. *Connectivity.* Table listing hatchling abundance, by species, for each nesting area and connectivity between those areas and the spill site. Percentage of particles backtracked from the spill site arriving from each of the nesting areas of green, loggerhead, and Kemp's ridley turtles within a given year, scaled relative to the number of particles that encountered any nesting area during that same period.

Table S2. *Literature-Survival*. Table of data used for estimating clutch size (number of eggs per nest), clutch survival, and oceanic survival.

Table S3. *Green (number)*. The number of each age class of surface-pelagic green turtles from each of the twenty-four nesting areas estimated to be at the spill site based on median, maximum, and minimum estimates of oceanic survival.

Table S4. *Loggerhead (number)*. The number of each age class of surface-pelagic loggerhead turtles from each of the eight nesting areas estimated to be at the spill site based on median, maximum, and minimum estimates oceanic survival.

Table S5. *Kemp's ridley (number)*. The number of each age class of surface-pelagic Kemp's ridley turtles from each of the four nesting areas estimated to be at the spill site based on median, maximum, and minimum estimates of oceanic survival. Table includes results for passive drift "retentive behavior" simulations.

Table S6. *Green (percent)*. The percentage of survivors of each age class of oceanic-stage green turtles from each nesting area estimated to be at the spill site.

Table S7. *Loggerhead (percent)*. The percentage of survivors of each age class of oceanic-stage loggerhead turtles from each nesting area estimated to be at the spill.

Table S8. *Kemp's ridley (percent)*. The percentage of the total survivors of each age class of surface-pelagic Kemp's ridley turtles from each nesting area estimated to be at the spill site. Table includes results for passive drift "retentive behavior" simulations.
